# Supplementary material for: Minimizing the number of optimizations for efficient community dynamic flux balance analysis
Source: PLoS Comput Biol. 2020 Sep 29;16(9):e1007786. doi: 10.1371/journal.pcbi.1007786 (PMC7546477; doi:10.1371/journal.pcbi.1007786)
Supplement: S1 Text — Proof of the main lemma of the paper. (PDF) [file pcbi.1007786.s001.pdf]

S1 Text: Existence of desired optimal basis.

James D. Brunner[1*],Nicholas Chia[1]

1Department of Surgery, Center for Individualized Medicine Microbiome Program, Mayo Clinic, Rochester, MN, USA

* brunner.james@mayo.edu

Lemma 1. *For a linear program with the form given in Eq.* (12) *with a basic optimal solution* $\boldsymbol{w}$*, there exists a basic index set* $\mathcal{I}$ *such that Eq.* (18) *holds and* $\dot{\boldsymbol{w}}$ *is optimal over the possible choice of basic index sets for* $\boldsymbol{w}$*.*

Proof. For convenience, we now restate Eq. (12):

$$\left\{\begin{array}{c} \max(\tilde{\boldsymbol{\phi}} \cdot \tilde{\gamma}) \\ \begin{bmatrix}\tilde{A} & I\end{bmatrix}\begin{bmatrix}\tilde{\boldsymbol{\phi}} \\ \boldsymbol{s}\end{bmatrix} = \boldsymbol{c} \\ \tilde{\phi}_i \geq 0, s_i \geq 0 \end{array}\right\}$$

where we write $(\tilde{\boldsymbol{\phi}}, \boldsymbol{s}) = \boldsymbol{w}$.

We note that there is a finite number of basic index sets for $\boldsymbol{w}$, and so we need only show that there exists $\mathcal{I}$ such that Eq. (18) holds. Then, the existence of an optimal such $\mathcal{I}$ follows trivially.

If $\boldsymbol{w}$ is not degenerate, then the unique choice of basic index set $\mathcal{I}$ satisfies Eq. (18). To see this, simply note that if $\boldsymbol{w}$ is non-degenerate, then for every $i \in \mathcal{I}$, $w_i > 0$. Thus, Eq. (18) only includes non-negativity constraints on $\dot{w}_i$ if $i \notin \mathcal{I}$, and for any $i \notin \mathcal{I}$, $\dot{w}_i = 0$. Thus, the non-negativity constraints are enforced. The equality constraints are enforced by the definition of $\boldsymbol{w}_{\mathcal{I}}(\boldsymbol{a})$ given in Eq. (13), which implies that $[\tilde{A}\ I]\boldsymbol{w}_{\mathcal{I}}(\boldsymbol{a}) = \boldsymbol{a}$ for any vector $\boldsymbol{a} \in \mathbb{R}^n$.

In the case of a degenerate solution $\boldsymbol{w}$, we use the following procedure to choose a set of basic variables. Let $\mathcal{J} \subset \{1, ..., n\}$ be the indices of the n_1 slack variables such that $s_j = 0$ if $j \in \mathcal{J}$ (recalling that each s_i is a component of the vector $\boldsymbol{w}$). Then, let $\tilde{A}_{\mathcal{J}}$ be the matrix with rows m_j of $\tilde{A}$ for $j \in \mathcal{J}$. Next, let $\mathcal{J}^*$ be the indices of the n_2 non-slack variables such that $\phi_j = 0$ and $I_{\mathcal{J}^*}$ the corresponding rows of the identity matrix I. Notice that we now have that

$$M\tilde{\boldsymbol{\phi}} = \begin{bmatrix}\tilde{A}_{\mathcal{J}} \\ -I_{\mathcal{J}^*}\end{bmatrix}\tilde{\boldsymbol{\phi}} = \begin{bmatrix}\boldsymbol{c}_{\mathcal{J}} \\ \boldsymbol{0}\end{bmatrix}. \tag{1}$$

and that if $w_j = 0$ then either $j \in \mathcal{J}^*$ or $w_j = s_k$ where $k \in \mathcal{J}$ so that $\boldsymbol{m}_k \cdot \tilde{\boldsymbol{\phi}} = c_k$ (i.e. s_k is a slack variable and $s_k = 0$). Notice that because Eq. (12) has a bounded solution, then we can assume without loss of generality that if $M \in \mathbb{R}^{q \times r}$, then $rank(M) = r$ (i.e. M is full rank) because $\boldsymbol{w}$ must satisfy at least r linearly independent constraints. If this is not the case, then the problem can be projected onto a lower dimensional subspace.

Consider the linear program

$$\left\{\begin{array}{c} \max(\boldsymbol{y} \cdot \gamma) \\ \begin{bmatrix}M & I\end{bmatrix}\begin{bmatrix}\boldsymbol{y}_{\tilde{\boldsymbol{\phi}}} \\ \boldsymbol{y}_{\boldsymbol{s}}\end{bmatrix} = \begin{bmatrix}\frac{d}{dt}\boldsymbol{c}_{\mathcal{J}} \\ \boldsymbol{0}\end{bmatrix} \\ y_j \geq 0 \end{array}\right\}. \tag{2}$$

Assume that there is some basic optimal solution to Eq. (2) with a basic index set $\hat{\mathcal{I}}$ such that exactly r slack variables are non-basic, where again $r = |\boldsymbol{\phi}|$ is the rank of the matrix M. This implies that there are r linearly independent rows of M (which we index by $\mathcal{J}^\dagger$) which form an invertible matrix $\tilde{M}$ such that

$$\tilde{M}\boldsymbol{y}_{\tilde{\boldsymbol{\phi}}} = \begin{bmatrix} \frac{d}{dt}\boldsymbol{c}_{\mathcal{J}^\dagger} \\ \boldsymbol{0} \end{bmatrix} \tag{3}$$

and we can then determine $\boldsymbol{y}_{\boldsymbol{s}}$ by

$$\boldsymbol{y}_{\boldsymbol{s}} = \begin{bmatrix} \frac{d}{dt}\boldsymbol{c}_{\mathcal{J}} \\ \boldsymbol{0} \end{bmatrix} - M\boldsymbol{y}_{\tilde{\boldsymbol{\phi}}} \tag{4}$$

and note that each $(\boldsymbol{y}_{\boldsymbol{s}})_i \geq 0$. We now rewrite $\dot{\boldsymbol{w}} = (\dot{\boldsymbol{w}}_{\tilde{\boldsymbol{\phi}}}, \dot{\boldsymbol{w}}_{\boldsymbol{s}})$ from Eq. (18) and define $\dot{\boldsymbol{w}}_{\tilde{\boldsymbol{\phi}}} = \boldsymbol{y}_{\tilde{\boldsymbol{\phi}}}$ and

$$\dot{\boldsymbol{w}}_{\boldsymbol{s}} = \frac{d}{dt}\boldsymbol{c} - M\dot{\boldsymbol{w}}_{\tilde{\boldsymbol{\phi}}} \tag{5}$$

and conclude that this satisfies the constraints of Eq. (18). Next, we take $\tilde{\boldsymbol{\phi}}$ to be the unique solution to

$$\tilde{M}\tilde{\boldsymbol{\phi}} = \begin{bmatrix} \boldsymbol{c}_{\mathcal{J}^\dagger} \\ \boldsymbol{0} \end{bmatrix} \tag{6}$$

and $\boldsymbol{s} = \boldsymbol{c} - \tilde{A}\tilde{\boldsymbol{\phi}}$.

Finally, we take $\mathcal{I} = (\hat{\mathcal{I}} \setminus \mathcal{J}^*) \cup \mathcal{J}^c$ and note that this basis set enforces exactly the same r linearly independent constraints as $\tilde{M}$[1].

We now prove that there is some basic optimal solution to Eq. (2) with a basic index set $\hat{\mathcal{I}}$ such that exactly r slack variables are non-basic, where r is the rank of the matrix M.

First we note that for any basic optimal solution, if there are $r^* > r$ slack variables which are non-basic, then there are r^* rows of $B_{\hat{\mathcal{I}}}$ which are non-zero only in the columns of M. Therefore, $B_{\hat{\mathcal{I}}}$ is not invertible. We can conclude that the number of non-basic slack variables is at most r.

Next, suppose $\dot{\boldsymbol{w}}^*$ is a basic optimal solution with basis $\mathcal{I}^*$ such that there are $r^* < r$ slack variables which are non-basic.

We would like to assume that there are at least r slack variables s_k^* corresponding to r linearly independent constraints such that $s_k^* = 0$. Recall that $\tilde{A}$ was formed with repeated (negated) columns in order write the problem in standard form (the non-negativity bounds of Eq. (12) are artificial). Therefore, we can find some vector $\boldsymbol{x}$ in the kernel of the matrix formed by the rows of $\tilde{A}$ corresponding to zero slacks which also has $\boldsymbol{x} \cdot \boldsymbol{\gamma} = 0$. We can therefore find a vector $\boldsymbol{y}$ in the kernel of

$$\begin{bmatrix} \tilde{A}_{\mathcal{J}} & I & 0 \\ -I_{\mathcal{J}^*} & 0 & I \end{bmatrix}$$

which has $y_k = 0$ if $s_k = 0$ and $y_j \neq 0$ if $s_j \neq 0$ and s_j corresponds to a constraint that is not a linear combination of the constraints corresponding to the $s_k = 0$. There is at least one such constraint as long as the 0 slack variables correspond to constraints with span less than dimension r, and so we can take $\dot{\boldsymbol{w}} + \lambda\boldsymbol{y}$ for some λ and so increase the number of non-zero slack variables. We can therefore assume without loss of generality that there are at least r slack variables s_k^* corresponding to r linearly independent constraints such that $s_k^* = 0$, as was desired.

[1] In practice, we may simply use $\tilde{M}$ to find $\tilde{\boldsymbol{\phi}}$

We can finally choose some linearly independent set of r constraints which correspond to 0 slack variables, and call the matrix whose rows are these constraint vectors M^*. Now, because there are $r^* < r$ non-slack basic variables, there is some non-slack, non-basic variable v_j such that the column m_j^* of M^* (and m_j of M) is linearly independent from the columns corresponding to the r^* non-slack basic variables. We can conclude that if

$$B_{\mathcal{I}^*} \boldsymbol{\lambda} = m_j \tag{7}$$

then there is some $\lambda_k \neq 0$ where k corresponds to the index of a slack variable with $s_k = 0$. We can remove k from the basic index set and add j without changing $\dot{\boldsymbol{w}}^*$, and therefore preserving optimality and feasibility. We have then increased the number of non-basic slack variables, and we can repeat if necessary to form $\hat{\mathcal{I}}$ with exactly r non-basic slack variables.

□
