# Supplementary material for: Minimizing the number of optimizations for efficient community dynamic flux balance analysis
Source: PLoS Comput Biol. 2020 Sep 29;16(9):e1007786. doi: 10.1371/journal.pcbi.1007786 (PMC7546477; doi:10.1371/journal.pcbi.1007786)
Supplement: Attachment — Submitted filename: ResponsesToReviewers.docx [file pcbi.1007786.s004.docx]

Reviewer's Responses to Questions

**Comments to the Authors:
Please note here if the review is uploaded as an attachment.**

**Reviewer #1:** The authors propose a method for efficient dynamic flux balance analysis (DFBA) of community metabolic models that reduces the number of LP solutions as compared to brute-force methods based on solving the LP at each time step. The method is applied to an 8-species community to demonstrate its usefulness. While the development of numerical methods for community DFBA is an important problem, the manuscript has several shortcomings that need to be addressed before publication can be recommended.

1. First and foremost, the authors fail to explain why their method is superior to or even different from the DFBA method proposed by the Barton group at MIT [33, 34]. The two methods seem very similar as suggested by the authors:

"Hoeffner et al, [34], used this observation to introduce a variable step-size method for dynamic FBA. In that method a basic index set is chosen by adding biological constraints to the optimization problem hierarchically until a unique optimal solution is found. The challenge of such an approach is in choosing the basis for the optimal solution, as the optimal basis is not guaranteed to be unique. We describe how to choose a basis from among the possibilities provided from an FBA solution which is most likely to remain optimal as simulation proceeds forward. We therefore prioritize reducing the number of times the linear program must be solved."

So the claim innovation here is more rational choice of the optimal basis? This point is critical and not clearly explained.

Yes, the innovation is a more rational choice of optimal basis. We have clarified by edited the above as (line 155):

However, resolving the system at each time step is not necessary, as the solution the optimization problem at some initial time can actually be used to compute future optimal solutions. H\"{o}ffner et al., \cite{hoffner2012}, used this observation to introduce a variable step-size method for dynamic FBA. In that method a basic index set is chosen by adding biological constraints to the optimization problem hierarchically until a unique optimal flux vector is found. The challenge of such an approach is in choosing the basis for the optimal solution, as the optimal basis is not guaranteed to be unique **even for a unique optimal flux solution. In fact, due to the nature of the method of H\"{o}ffner et al. and of our method, any optimization past the initial solution that must be carried out is guaranteed to have a solution with a non-unique basis. Furthermore, many choices of optimal basis will not provide a solution for future time-steps, so that choosing among these bases must be done intelligently. Unfortunately, H\"{o}ffner et al. \cite{hoffner2012} do not provide a method for choosing among non-unique bases for a single linear program solution.**

Our method seeks to solve this problem by choosing a basis from among the possibilities provided from an FBA solution which is most likely to remain optimal as simulation proceeds forward. We therefore prioritize reducing the number of times the linear program must be solved, **choosing our basis based on the mathematical properties of the system which gives the best chance of providing a solution at future time-steps.**

2. While the authors are correct that the COBRA toolbox offers a simple and inefficient Euler-based method (e.g. [30]), I would hardly consider this method to be state-of-the-art. If the authors would like to claim superiority, then they should compare their method to the DFBA method proposed by the Barton group [33, 34].

We now compare directly to the Barton group, using as our metric the number of optimizations required. To do this, we use the implementation recently published by Prof. David Tourigny for the case of single organisms, and the implementation DFBAlab from the Barton group for communities. We note that DFBAlab seems to have some numerical error resulting in negative maximum growth rates when metabolites have been nearly depleted. In one simulation, DFBAlab was unable to simulate past time t=3.034277 as it could not find an optimal basis for the LP problem.

3. When comparing methods, the authors state that the Euler-type DFBA method required a very short time step to ensure non-negative metabolite concentrations. Perhaps a more efficient implementation of the Euler method would be to utilize an ODE solution method that ensure non-negativity of the dependent variables, such as the ODE solvers available in MATLAB. Did the authors consider this option, which would allow a fairer comparison?

We now compare not with the basic Euler method but instead with more sophisticated so called “direct methods” which make use of the 5 available ODE integration methods available in the SciPy computational library. These methods employ adaptive step sizes. We demonstrate that our method requires far fewer optimizations than the direct method.

4. The application to the 8-species community is a reasonable test of the method, but some biological context also would be useful. For example, why focus on uracil as a crossfed metabolite? Why assume linear uptake kinetics rather than the usual Michael-Menten kinetics?

We did not intend to focus on any particular biological situation or observation, and so for clarity we have removed this example from the paper. We assume linear uptake parameters only for computational simplicity, and mention in the paper that other choices may be more realistic.

5. A computation time of over 15 minutes for a 1 hour simulation (see Fig. 5) does not seem particularly fast to me. Please compare computation times with a state-of-the-art DFBA code.

We have replaced consideration of computational time with a count of the number of optimizations required. We believe this is a more “apples to apples” comparison, as state-of-the-art DFBA code benefits from optimally designed code while employing a less efficient fundamental algorithm at its core. By comparing with direct methods on the basis of number of optimizations, we are comparing the core methods rather than the quality of implementation.

We now explain in the manuscript the following (line 342):

We can compare the efficiency of \cref{brunner} with modern dynamic FBA methods by counting the number of times a large linear program must be carried out over the course of a simulation. At their core, state-of-art dynamic FBA tools such as \emph{d-OptCom} \cite{zomorrodi2014} and \emph{COMETS} \cite{harcombe2014} employ the direct method of calling an ODE-solving method with the linear program set as the right-hand-side. In the case of Euler's method, the resulting ODE can be integrated by hand between time-steps. This last strategy is often referred to as the ``static optimization approach" \cite{hoffner2012}.

We compared simulation of various combinations of the organisms \emph{Escherichia coli str. K-12 substr. MG1655} (model iJR904), \emph{Saccharomyces cerevisiae S288C} (model iND705), \emph{Pseudomonas putida KT2440} (model iJN746) and \emph{Mycobacterium tuberculosis H37Rv} (model iEK1008), using models from the BiGG database \cite{king2016bigg} (see \nameref{models} for details). We counted the optimizations required for our model, as well as for direct methods using the numerical ODE solvers \emph{vode}, \emph{zvode}, \emph{lsoda}, \emph{dopri5}, and \emph{dop853} from the SciPy library. All of these numerical ODE solvers use adaptive step sizes for accuracy and stability, and so represent optimized choices of time-steps. Additionally, we compared the method of H\"{o}ffner et al. as implimented in the MatLab package \emph{DFBAlab} \cite{gomez2014dfbalab}.

For our method and the direct method, we allowed exchange of every metabolite detailed in \nameref{media} with initial metabolite concentrations given by that same file, and with initial biomass of $0.3$ for each species. The file \verb|sim_comm.py| in the supplementary repository \nameref{software} contains complete simulation set-up.

To compare with the method of H\"{o}ffner et al. \cite{hoffner2012}, we use the newly available Python package from the research group of Dr. David Tourigny titled \emph{dynamic-fba} \cite{dfbaNew} for single organisms. This package allows simulation without secondary optimizations, as our does, and so is more similar to our prototype tool for comparison. Unfortunately, this package is currently only able to simulate single organisms at the time of publishing. For microbial communities, we can compare with the MatLab package DFBAlab \cite{gomez2014dfbalab} which requires all dynamics variables to be optimized in a secondary optimization. For simulations with DFBAlab, we use only the low-concentration metabolites D-glucose, oxygen, and cob(I)alamin from the M9 medium detailed in \nameref{media} as dynamically varying metabolites. It is worth noting that these are the most favorable conditions we could find for the method of H"{o}ffner \cite{hoffner2012,gomez2014dfbalab} et al. which are still biologically equivalent to our other simulations.

6. Several of the plots lack axes labels (e.g. Fig. 6, 7 and 9). Please correct.

All plots are now fully labeled.

**Reviewer #2:** In this manuscript, Brunner and Chia have proposed an algorithm to accelerate the simulation of time-course behavior of microbial communities. The existing dFBA algorithm was improved upon by implementing a solution-hopping strategy in order to reduce the number of times the FBA problem must be solved at each time step. The authors have shown that this approach remains scalable for communities containing up to 8 members when compared to the classic dFBA algorithm. Overall, this speed-up is significant and when properly benchmarked against existing state-of-the-art algorithms, has the potential to be an important advancement in the field of microbial community modeling.

Major comments:

1. The authors have only compared their algorithm against the classical dFBA. However, in recent years, several meaningful algorithms have been developed such as d-OptCom (Zomorrodi and Islam et al, ACS Synthetic Biology, 2014), COMETS (Harcombe et al, Cell Rep., 2014), and SteadyCom (Chan et al, PLoS Comput Biol, 2017). The authors must benchmark their method in terms of both time and performance with the above methods.

We now make clear that our method represents a fundamentally different method of solution from all available solvers, with the closest comparable the method of Barton. We clarify in the manuscript as follows (line 145):

In addition to Euler's method, more sophisticated ODE solvers may be used in the so-called ``direct" method of simply recomputing the FBA optimization at every time-step. This can provide better solution accuracy and potentially larger time-steps, but may also require more than one FBA optimization at each time-step. For instance, the Runge-Kutta fourth order method \cite{bradie} requires four FBA solutions at each time step. Direct methods are implemented in the COBRA toolbox \cite{cobratoolbox2017} and are the central algorithm in many modern tools, including those of Zhuang et al. \cite{zhuang2011genome,zhuang2012design}, Harcobe et al. \cite{harcombe2014}, Zomorrodi et al. \cite{zomorrodi2014}, Louca and Doebeli \cite{louca2015calibration}, and Popp and Centler \cite{popp2019mubialsim}. Notably, any direct method requires at least one complete recalculation of the network fluxes \emph{at each time-step}.

2. The proposed method assumes that maximization of total biomass is the community objective. A consequence of this is that only the fastest growing organism will show any growth over time and cause the community to become a monoculture. The authors must comment on how their method safeguards against such outcomes.

We now clarify in the manuscript (line 212):

[E]ach optimization is carried out in a manner that depends only the status of the metabolite pool and is independent from the optimizations of other organisms. There is therefore no shared ``community objective". Instead, each organism optimizes according to only its own internal objective.

3. The classical dFBA algorithm consists of a numerical ODE integration wrapped around an instantaneous FBA problem. It is the requirement of 10,000 integration steps that leads to the high computational cost. As a result of this, the reported “85% reduction in computation time” may be inflated. It would be more meaningful to compare the two algorithms using an adaptive time-step based integration such as adaptive Runge-Kutta or Richardson’s extrapolation, or a faster ODE solver such as CVODE from the SUNDIALS package.

We now compare with 5 modern ODE solver methods from the SciPy toolbox. Please see our response to reviewer 1 for details.

4. Because the forward simulation method approximates the optimal FBA solution at multiple time steps, the authors must also compare the accuracy of their simulations with that of classical dFBA and comment on the accuracy vs. speed trade-off of their proposed algorithm.

We thank the reviewer for pointing this out. In the manuscript, we clarify by noting (line 377):

Our method provides much less theoretical error in dynamic FBA solutions than traditional methods. In fact, \Cref{brunner} implies that a simulation of a microbial community can be divided into time intervals on which the algorithm is exact. Of course, this assumes that the linear ODE solved in these intervals is solved exactly rather than numerically.

This allows us to highlight one of the strengths of this approach, which is that we can produce exact answers with no trade-off in accuracy.

5. Metabolic fluxes have a large dynamic range of values spanning up to three orders of magnitude. This can often lead to numerical instability issues where concentrations assume negative values. The authors must justify why 10,000 time-steps are sufficient to bypass this issue.

The ODE solver methods that we now compare with contain internal sub-routines to estimate numerical stability and adjust time-steps accordingly.

6. The authors have stated that re-solving is necessary when the solution becomes infeasible. However, metabolic networks are carefully constructed such that, as long as concentration of species in the extracellular media remain non-negative, the FBA problem will never be infeasible. The authors must clarify what leads to infeasibility in their FBA problem. Could this be an artefact of the approximation introduced in this algorithm to bypass re-solving the FBA problem?

Our method requires re-solving not when the *problem* becomes infeasible but instead when the *solution we have been following* becomes infeasible. We have clarified as follows (in the abstract):

(i.e. the solution to **the linear system of equations** remains a feasible **to the linear program**)

(line # 268):

We then use this same system to simulate forward without the need to re-solve the **solution to the system of equations until there is no longer a feasible solution to the linear program.**

Finally, in Example 1, we include the following:

We may follow $\b{w}_{\cI_2}$ and be insured of remaining at an optimal solution to the linear program until $t = 20 + \varepsilon$, at which point $\b{w}_{\cI_2} = (10,-\varepsilon/2,0,10,0)$, which is not a feasible solution to the linear program. At time $t=20$, a re-optimization is required to choose a new basis.

which illustrates the method.

7. The authors must also include a discussion on the new analyses enabled by the accelerated simulation of microbial communities. In particular, what previously difficult to answer questions can now be addressed by the community due to the advancement afforded by this proposed method.

Two of the included examples were intended for this purpose. We now include the following discussion as preface to these examples (line 434):

There has been a recent surge in interest in modeling microbial communities using genome-scale metabolic models, much of which has focused on equilibrium methods \cite{gottstein2016constraint,Diener2018,Hale2018,islam2019metabolic,koch2019redcom}. In order to capture transient behavior and dynamic responses to stimuli, dynamic FBA has also been applied to microbial communities \cite{zomorrodi2014,xu2019modeling,succurro2019emergent}. However, community dynamic FBA invariable leads to a large dynamical system with a high-dimensional parameter space, often with little to know knowledge of parameter values. Any parameter fitting therefore requires repeated numerical simulation of the system. Existing tools to do this are built around a direct simulation approach, requiring many linear program solutions. By drastically reducing the number of optimizations required for numerical simulation, our approach offers the promise of efficient numerical simulation of dynamic FBA which will make parameter fitting more tractable, and may even allow conclusions without well-fit parameters.

Below, we demonstrate that the problem of parameter fitting is an important one by show that experimental outcome in even small communities is sensitive to changes in kinetic parameters. Precisely, the kinetic parameters governing the uptake rate of nutrients (i.e., the parameters of the functions $\b{c}^2_i$ in \cref{FBA_summary}) have a profound effect on species competition.

Next, we show how repeated simulation with randomly sampled parameters can provide some insight into community structure even without a well-fit set of nutrient uptake parameters. These examples demonstrate the importance of efficient dynamic FBA to microbial community modeling.

Minor Comments:

1. The authors must clarify that their method improves upon the Static Optimization Approach of dFBA.

We now clarify the following (347):

In the case of Euler's method, the resulting ODE can be integrated by hand between time-steps. This last strategy is often referred to as the ``static optimization approach" \cite{hoffner2012}.

and also discuss how our method improves upon any direct method, including this one.

2. In the formulation shown in Equation (4), the quantity “c” is used prior to its definition. Furthermore, the function c(y) has not been defined in this manuscript.

We clarify as follows (187):

where $\b{c}^1_i$ is some vector of lower flux bounds while $\b{c}^2_i(\b{y})$ is some vector-valued function of the available metabolites which represents upper flux bounds.

3. It is recommended that different subscripts be used to denote microbial species, metabolites, and reactions for clarity of presentation. The same subscript “i” has been used to represent all of these quantities.

We use “i” to denote microbial species and “j” to denote metabolites, and have edited to insure this consistent.

4. In equations (6), (7), and (8), it is unclear why all fluxes are represented in terms of intracellular fluxes only. Generally, the kernel of the stoichiometry matrix is represented in terms of all but one extracellular exchange fluxes, and the intracellular fluxes causing a rank-deficiency in the stoichiometry matrix.

We thank the reviewer for pointing this out. We now clarify this unique notational choice in the preceding paragraph (and equation 5). The choice is made to emphasize that internal balancing is what ultimately determines the external reactions. To this end, we note in the manuscript (line 200):

Making this change in notation allows us to see that the optimization problem of flux balance analysis is essentially internal to the cell, with external reactions providing constraints.

We believe this choice helps us to highlight one advantage of our approach – the lack of requirement for a “community-wide” objective function.

**Reviewer #3:** The authors present a new algorithm (surfinFBA) for computing dynamic FBA simulations which does not require solving the linear problem at every time step, which greatly improves the computation times. Although the method looks promising, the quality of the manuscript is not so great and, in my point of view, needs substantial restructuring.

Major issues:

* The main emphasis of the paper is put on the mathematical details of deriving the algorithm, which would be interesting in a mathematical / computer science journal, but is not so interesting to the target audience of this journal. I would suggest moving these details to a supplementary text, and focusing the main text on the simulation results and benchmark of the new method.

The authors have consulted with the journal editors about the appropriateness of the paper for the journal. We note the following in that correspondence:

We would like to note that Reviewer #3 is indeed correct that the emphasis of this paper is on the mathematical details of the algorithm and not on the performance of a software package. More concerning, however, is the notion that PLOS CompBio is therefore the wrong venue for publishing work related to computational biology algorithms. I have been a longstanding member of the computational biology community, and would note that computational biology conferences with computer-science-like proceedings, such as those from RECOMB, are often published in PLOS CompBio. The work we submitted is targeting a similar audience, which has historically emphasized mathematical derivations of algorithms. It is upon this basis that we submitted our work to PLOS CompBio.

…

We think editing the manuscript to clarify scope would significantly improve the manuscript, highlight our specific contributions to the scientific community, and limit the apparent disappointment that we did not go head-to-head with tools that all use the same core Euler method (some mentioned by reviewers are DyMMM, COMETS and dOptCom).

We also note that the proof of Lemma 1 has been moved to the appendix to facilitate streamlined reading of the manuscript.

* The authors present the classic and new algorithm as Figures in the manuscript, and throughout the text often refer to running these algorithms as implementing / simulating Fig.1 and Fig. 3. I find this inappropriate and very confusing. The two algorithms should be presented as methods in the Methods section and referred to by their names, not by figure numbers.

The new algorithm is now presented in the methods section.

* The authors cite the first dFBA method by Varma and Palsson (1994), but they should also mention the first implementation of dFBA for multi-species simulation, the DyMMM method by Zhuang and co-workers (2011).

We now include this reference.

* There are also more recent multi-species dFBA methods like MCM (Louca and Doebeli, 2015) and microbialSim (Popp and Centler, bioRxiv, 2019). The authors should also compare the advantages / disadvantages of their method with these more recent methods. For instance, in their pre-print, Popp and Centler show that microbialSim as able to simulate a 773-species community, a much larger size than the 8-species community simulated with surfinFBA.

We have clarified throughout the manuscript that our method is a fundamentally different method of solution than available solvers, with the closest method being the method of Barton. Therefore, we do not feel it is appropriate to compare as *computation tools* but instead as *theoretical methods.*

Please see our response to reviewer 1 for specifics.

* The authors should better classify how they setup the simulation conditions. In the paper they mention they use the "COBRApy default simulated medium". This does not make sense. COBRApy is just a simulation library, it makes no assumptions regarding the medium. The medium is something the user must decide upon when parameterizing a model.

We now include details of the medium used in the supplemental files, and more simulation details in the supplemental github repository.

Minor issues:

* The authors mention they downloaded the models from the PATRIC database, which as far as I know is a genome database, not a model database, and I could not find how to download an SBML model from PATRIC. The models provided in the github repository are JSON files with modelSEED annotations, so I supposed they were built with modelSEED. This should be better clarified.

We have switched to using models from the BiGG database, URLs are provided in the supplemental materials.

**Reviewer #4:** We have read “Minimizing he number of optimizations for efficient community dynamic flux balance analysis” by Brunner and Chia. The authors have developed a method named surfinFBA for performing dynamic flux balance analysis calculations for in-silico communities of organisms that reduces the number of optimization problems that must be solved. This approach leads to an 85% reduction in compute time required relative to the standard method implemented in the COBRA toolbox (a package frequently used by the genome-scale modelling community). We applaud the authors use of github to disseminate the method in a usable format. Our interest in this manuscript is only tempered by a lack of discussion, and insufficient benchmarking relative to the classical method performed by the authors.
Major comments
1. The author demonstrates that surfinFBA provides a 85% speed increase in dynamic FBA simulations relative to the standard method. Supplementary File 1 compares surfinFBA to the classic method implemented in the COBRA toolbox. We appreciate seeing the time differences however there is no comparison of results between the two implementations. The author must add an analysis demonstrating the results are equivalent between the two tools, otherwise the time differences is less meaningful. Adding computed dFBA profiles from both the classic method and the novel method to the supplementary file for comparison would be a first step.

We would like to thank the reviewer for this suggestion. In order to compare tools, we now compute solutions for 4 single organisms and compute the differences in the solutions computed. Furthermore, we note that solutions to flux balance analysis (and therefore dynamic flux balance analysis) are not always unique. Therefore, differences in simulations between methods are not necessarily due to any drawback in either method. We clarify in the manuscript (line 425):

We note that discrepancies in dynamic FBA simulation may not always be due to numerical error, but instead due to non-uniqueness in optimal flux solutions. Our method provides a strategy for choosing between non-unique representations (in the form of a basis) of a single optimal flux solution. The method of H\"{o}ffner et al. \cite{hoffner2012} does provide a lexicographic strategy for choosing between non-unique optimal flux solutions based on biological, rather than mathematical, considerations. We note that for complete reproducibility, our method should be integrated with some biologically based strategy for choosing between non-unique optima.

Additionally, we discuss the error of the solution in detail (see response to reviewer 2 point 4).

2. A key value of FBA and dFBA simulations is their mechanistic nature enabling interpretation and contextualization of results. Thus, we were disappointed in the statement on line 436 where the authors mention that P. veronii had “some competitive advantage” in the default media. What media, specifically was used? Models usually come pre-loaded with a default media, but the COBRA toolbox itself does not (this should be corrected in the manuscript). The author should utilize the simulation results to trace the media component providing this competitive advantage to P. veronii. What aspect of the metabolic network for P. veronnii provides this competitive advantage? A deeper dive into some of the results presented here would provide more value to the manuscript.

We have corrected reference to medium and now include details of the medium simulated as a supplemental file. Additionally, we have removed mention of specific biological results - such as the competitive advantage of P. veronii – because these are no longer truly in the spirit of the manuscript. We discuss the need for efficient dFBA, and in fact argue that without it any conclusions cannot be as powerful as one would like. We have clarified that our method represents an improved method, rather than an already faster tool.

3. The manuscript ends rather abruptly, and we were disappointed in the lack of discussion section. Now that dFBA simulations can be run more efficiently what applications do the authors envision? The applications presented at the end are informative but additional discussion of their value would be illuminating.

A short conclusion section has been added.

4. Please provide details of all simulations performed. The stoichiometric matrices and flux bounds used should be provided in an excel sheet

Media files and model URLs are now available as supplemental files, as well as all code in the github repository.

Minor comments
1. Be sure to distinguish between the python and matlab implementations of the cobra toolbox – I believe all references are to the python implementation but please make this explicit. Also reference the version used for benchmarking.

We clarify throughout that all comparisons are done with python packages with the exception of DFBAlab, the only MatLab packaged used.

2. Be sure to label axes in all figures – e.g. 6 and 7.

All figure axes are now labeled, with the exception of mathematical cartoons which are included to illustrate the mathematical concepts (fig 1).
